# Hybrid immunity in older adults is associated with reduced SARS-CoV-2 infections following BNT162b2 COVID-19 immunisation

**DOI:** 10.1038/s43856-023-00303-y

**Published:** 2023-06-16

**Authors:** Scott J. C. Pallett, Joseph Heskin, Fergus Keating, Andrea Mazzella, Hannah Taylor, Aatish Patel, Georgia Lamb, Deborah Sturdy, Natalie Eisler, Sarah Denny, Esmita Charani, Paul Randell, Nabeela Mughal, Eleanor Parker, Carolina Rosadas de Oliveira, Michael Rayment, Rachael Jones, Richard Tedder, Myra McClure, Elisabetta Groppelli, Gary W. Davies, Matthew K. O’Shea, Luke S. P. Moore

**Affiliations:** 1 Clinical Infection Department, Chelsea and Westminster NHS Foundation Trust, London, UK; 2 Centre of Defence Pathology, Royal Centre for Defence Medicine, Queen Elizabeth Hospital Birmingham, Birmingham, UK; 3 Royal Hospital Chelsea, Royal Hospital Road, London, UK; 4 Institute for Infection and Immunity, St George’s University of London, London, UK; 5 Army Health Branch, Army Headquarters, Andover, UK; 6 Chief Nurse, Adult Social Care, UK Department of Health and Social Care, London, UK; 7 North West London Pathology, London, UK; 8 Department of Infectious Disease, Faculty of Medicine, Imperial College London, London, UK; 9 Institute of Immunology and Immunotherapy, College of Medical & Dental Sciences, University of Birmingham, Birmingham, UK; 10 Imperial College London, NIHR Health Protection Research Unit in Healthcare Associated Infections and Antimicrobial Resistance, London, UK

**Keywords:** RNA vaccines, SARS-CoV-2, ELISA, Ageing

## Abstract

**Background:**

Older adults, particularly in long-term care facilities (LTCF), remain at considerable risk from SARS-CoV-2. Data on the protective effect and mechanisms of hybrid immunity are skewed towards young adults precluding targeted vaccination strategies.

**Methods:**

A single-centre longitudinal seroprevalence vaccine response study was conducted with 280 LCTF participants (median 82 yrs, IQR 76-88 yrs; 95.4% male). Screening by SARS-CoV-2 polymerase chain reaction with weekly asymptomatic/symptomatic testing (March 2020-October 2021) and serology pre-/post-two-dose Pfizer-BioNTech BNT162b2 vaccination for (i) anti-nucleocapsid, (ii) quantified anti-receptor binding domain (RBD) antibodies at three time-intervals, (iii) pseudovirus neutralisation, and (iv) inhibition by anti-RBD competitive ELISA were conducted. Neutralisation activity: antibody titre relationship was assessed via beta linear-log regression and RBD antibody-binding inhibition: post-vaccine infection relationship by Wilcoxon rank sum test.

**Results:**

Here we show neutralising antibody titres are 9.2-fold (95% CI 5.8–14.5) higher associated with hybrid immunity (*p* < 0.00001); +7.5-fold (95% CI 4.6-12.1) with asymptomatic infection; +20.3-fold, 95% (CI 9.7-42.5) with symptomatic infection. A strong association is observed between antibody titre: neutralising activity (*p* < 0.00001) and rising anti-RBD antibody titre: RBD antibody-binding inhibition (*p* < 0.001), although 18/169 (10.7%) participants with high anti-RBD titre (>100BAU/ml), show inhibition <75%. Higher RBD antibody-binding inhibition values are associated with hybrid immunity and reduced likelihood of infection (*p* = 0.003).

**Conclusions:**

Hybrid immunity in older adults was associated with considerably higher antibody titres, neutralisation and inhibition capacity. Instances of high anti-RBD titre with lower inhibition suggests antibody quantity and quality as independent potential correlates of protection, highlighting added value of measuring inhibition over antibody titre alone to inform vaccine strategy.

## Plain language summary

Older adults continue to be at risk of COVID-19, particularly in residential care home settings. We investigated the effect of infection and vaccination on antibody development and subsequent SARS-CoV-2 infection in older adults. Antibodies are proteins that the immune system produces on infection or vaccination that can help respond to subsequent infection with SARS-CoV-2. We found that older adults produce antibodies to SARS-CoV-2 after 2-doses of Pfizer BioNTech BNT162b2 vaccine. The strongest immune responses were seen among those older adults who also had prior history of infection. The results highlight the importance of both antibody quality and quantity when considering possible indicators of protection against COVID-19 and supports the need for a third, booster, vaccination in this age group..

## Abstract (web summary)

Pallett, Heskin et al. monitor longitudinal response in older adults following vaccination with the BNT162b2 COVID-19 (Pfizer-BioNTech) vaccination. Hybrid immunity is associated with higher antibody titres, neutralisation and inhibition capacity.

## Introduction

Severe acute respiratory syndrome coronavirus-2 (SARS-CoV-2) represents a considerable risk to older adults and those with multiple co-morbidities^1–3^. High morbidity and mortality rates following SARS-CoV-2 infection during the early stages of the pandemic disproportionately affected older adults, particularly those in long-term care facilities (LTCF)^4,5^, and concern has been raised over age-related immune response heterogeneity^6–8^. Recent data has suggested vaccine prevention of death after hospitalisation for severe SARS-CoV-2 infection was up to 22.6% lower in adults over 80 years old^9^.

Neutralising antibody titres are thought to be predictive of protection against severe infection^10^. Reduced neutralising activity against emergent variants of concern (VOC) has been recognised, however^10^, including in older adults^11^. Additionally, a recent longitudinal study has suggested no direct association between the measurement of titres and neutralising capacity^12^. While hybrid immunity (prior infection and vaccination) in younger adults has been shown to provide improved protection against severe infection, including against the Omicron variant, older adults are a less well-described yet key at-risk group^13^. Indeed, an absence of sufficient data in older adults is highlighted as precluding any possibility of considering targeted vaccination strategies in those at high risk of severe infection, of which LCTF residents and those over 80 years make up the first two priority groups^13,14^. Efficacy studies to date, for example, have leaned heavily on recruitment of healthcare workers or specific high-risk immune-suppressed patients^15^, while the majority of larger population-based data collection initiatives tend to be skewed towards younger adults^16^. Where emerging neutralisation data in older-adult populations is becoming available, studies have been limited by a combination of small sample size and/or reduced confidence in reliably ruling out virus exposure prior to vaccination^8^. The ability of older adults to mount comparable responses, and the importance of the interplay between natural infection and vaccination in this group, remain less clear.

Across Europe, individuals living in LTCFs have experienced a serious toll from the SARS-CoV-2 pandemic^5^. It is vital that we better understand the functional immune response to natural infection and vaccination in this high-risk population in order to inform targeted public health policy and vaccine strategy, including identification of priority groups for vaccine booster(s). We report here findings of a longitudinal seroprevalence study at an LTCF, the Royal Hospital Chelsea (RHC), enhanced by high-fidelity, regular SARS-CoV-2 screening and availability of highly granular prior exposure and infection outcome data provided throughout. The study aims to examine the ability of older adults to mount a robust immune response to Pfizer-BioNTech BNT162b2 vaccination, finding an association between antibody-neutralising titres and hybrid immunity, with strong evidence for an association between rising antibody titres and both neutralisation and RBD antibody-binding inhibition capacity.

## Methods

A single centre prospective longitudinal seroprevalence study was conducted at the Royal Hospital Chelsea (RHC), London, UK, between 22 March 2020 and 04 October 2021 (REC Ref.22/EE/0083). The primary study objective was to explore the ability of an older-adult population to mount robust immune responses in response to a two-dose Pfizer-BioNTech BNT162b2 vaccination regimen and the effect of prior infection. Where antibody quantity and quality are independent measures that may both respond to immunisation, the immune response was assessed by measuring a change in antibody titre, neutralising capacity and RBD antibody-binding inhibition. Secondary objectives were to (i) assess for waning of a variety of SARS-CoV-2 specific antibodies over time and (ii) assess the relationship of any breakthrough infection in the context of antibody response; specific to an elderly population.

### Setting

The RHC is a 290-bedded combined residential and nursing home with an on-site infirmary and embedded primary care service. Social-distancing measures, including site lockdown, were instigated from 19 March 2020, with the facility remaining closed to non-essential visitors until UK Government’s social-distancing policy change on 19 July 21. Inclusion criteria were: all RHC residents throughout the study period able to provide written informed consent were eligible for the study. Exclusion criteria were: those unable to provide written informed consent, prior recruitment to the UK Health Security Agency supported Sarscov2 Immunity & REinfection EvaluatioN (SIREN) study or receipt of a vaccine other than the Pfizer-BioNTech BNT162b2. Throughout the pandemic period, Consultant-led secondary care advice (elderly care, respiratory, microbiology and infectious diseases) were provided by Chelsea and Westminster NHS Foundation Trust, London, UK. Public Health and infection and prevention control (IPC) advice were provided by the Army’s Senior Health Advisor team. On-site clinical provision was bolstered during periods of high community transmission by an Army junior doctor and six combat medical technicians. Admission to secondary care, where required, was to Chelsea and Westminster Hospital (CWH). Data on prior conditions or medications associated with immunosuppression and those with chronic respiratory disease were collected^17^.

### SARS-CoV-2 polymerase chain reaction (PCR) testing

Participants underwent routine weekly screening via SARS-CoV-2 PCR of nasopharyngeal swabs with additional acute phase swabbing of symptomatic cases/contacts tested as required. Participants were established as symptomatic based on UK guidance published by the UK Health Security Agency (including a new continuous cough, fever, loss of taste/smell, unexplained lethargy, myalgia, anorexia, unexplained headaches, sore throat, diarrhoea and/or vomiting^18^. Patients were screened for PCR testing by onsite medical and/or nursing staff available across the site from the end of March 2020. Asymptomatic infection was defined as new anti-NP seroconversion and/or new positive PCR result but exhibiting none of the above symptoms on clinical review. The diagnostic laboratory used a variety of platforms to undertake testing, depending on consumable supply chains. These were all CE-marked systems and had additionally undergone local validation and verification. SARS-CoV-2 PCR assays included those from AusDiagnostics (AusDiagnostics, Sydney, Australia), Thermo Fisher (Thermo Fisher Scientific, MA, USA) and Roche (Roche Holding AG, Basel, Switzerland).

### SARS-CoV-2 serological testing

Participants were invited to undertake blood draws for assessment of SARS-CoV-2 antibodies at three intervals, namely (i) as part of an initial outbreak investigation and coinciding with the end of the UK’s first wave of infection and relaxation of initial social distancing regulations (June 2020) and (ii) prior to receipt of the Pfizer-BioNTech BNT162b2 vaccine (December 2020) and (iii) 4 weeks after the second dose of Pfizer-BioNTech BNT162b2 vaccine (April 2021). Antibody waned over 8 months in older adults (using the same assay, see below)^19^ informed the decision to test at least once, but not more than, every 6 months for evidence of new seroconversion, while not unnecessarily increasing the sampling burden to participants. Likewise, in order to reduce the test burden on participants, maximise study retention and be in line with the UK vaccine strategy for an initial two-vaccine regimen, a separate blood draw after a single vaccine dose was not carried out.

Antibodies to the SARS-CoV-2 nucleocapsid protein (anti-NP) may be detected in serum following infection but not in response to immunisation. Assessment for evidence of seroconversion following SARS-CoV-2 infection (symptomatic or asymptomatic) was via an anti-nucleocapsid (anti-NP) IgG chemiluminescent microparticle immunoassay (CMIA) as per manufacture instructions (Abbot Laboratories, Lake Bluff, IL, USA). Anti-receptor binding domain (anti-RBD) antibodies have been previously shown to be predictive of neutralising response, and may be detectable following infection or immunisation^20^. Samples were therefore also tested on the commercial quantitative Abbott Architect IgG Quant II CMIA (Abbott IgG CMIA; Abbot Laboratories, Lake Bluff, IL, USA) in order to observe comparison with a commercially available assay marketed to assess SARS-CoV-2 antibodies, including neutralising antibodies. The Abbott IgG CMIA is a two-step automated immunoassay for qualitative and quantitative assessment targeting the S1 subunit of the RBD. Abbott arbitrary units (AU/ml) are multiplied by a factor of 0.142, giving a binding antibody unit (BAU)/ml result, providing a correlation with the WHO international standards^21^. The assay recognises BAU/ml >900 to be associated with high-neutralising capacity and the cut-off was considered as a further measure of change when comparing antibody titres pre- and post-vaccine delivery. In order to further characterise the response to prior SARS-CoV-2 infection and vaccination and provide a comparison with the competitive anti-RBD enzyme-linked immunosorbent assay described below, paired serum samples pre- and post-vaccine were measured using a quantitative hybrid in-house anti-RBD double antigen-binding assay (DABA) (Imperial College, London, UK)^22^. The RBD DABA utilises S1 antigen manipulated to express stably RBD epitopes and enable highly specific capture of anti-RBD antibody, detected by labelled fluid phase horseradish peroxidase-coupled RBD. RBD DABA binding ratios are converted and reported in binding antibody units/ml (BAU/ml), allowing correlation with World Health Organisation (WHO) international standards for SARS-CoV-2 IgG binding antibody units.

### Pseudotype virus neutralisation

Post-vaccine in vitro antibody neutralisation activity was further explored by pseudotype virus assay, with a comparison of results between SARS-CoV-2 infection naïve individuals and those with prior evidence of SARS-CoV-2 infection (both asymptomatic and symptomatic). A lentivirus that encodes for luciferase and pseudo-typed with the spike of SARS-CoV-2 was utilised. In brief, 16 µl participant sera was heat-inactivated (56 ^o^C, 30 min) and initial dilutions made with 5 µl HI serum and 2% growth medium (Dulbecco’s Modified Eagle Medium, penicillin/streptomycin, 2% fetal bovine serum [FBS]). A total of eight serial dilutions were made and mixed with PsV (0.5–1 × 10^6^ relative luminescence units/well) and incubated at 37 ^o^C for 48 h. Luciferase was then measured at 48 h post-infection (BrightGlo, Promega). All positive samples were reactive at 1:40 dilution. Using calculated logarithmic dilution factors, data were then able to be expressed as a percentage neutralisation, e.g. 100% meaning serum neutralised 100% of the PsV, as previously described by Ferrera and Temperton^23^.

### Competitive ELISA/inhibition assay

An in-house competitive anti-RBD antibody one-step competitive enzyme-linked immunosorbent assay was utilised to assess RBD antibody-binding inhibition. As part of the consent process, participants were asked for permission to utilise any remaining serum taken for serological and pseudovirus neutralisation testing on additional SARS-CoV-2 antibody assays as they became available. Those individuals that had sufficient paired samples remaining from pre- and post-vaccine sampling and had given permission were also tested by inhibition assay at these time points. The SARS-CoV-2 competitive ELISA methodology utilised was similar to those previously described in refs. ^24,25^. In brief, solid phase 96-microwell plates (NUNC Immunomodule, U8 Maxisorp wells) were coated with 100 μl of S1 antigen at a concentration of 5 μg/mL (MicroImmune Coating Buffer; ClinTech, Guildford, UK) overnight at 2−8 °C, followed by 3 h at 35−37 °C (under moist conditions) and 1 h at room temperature. Plates were washed once with 0.05% Tween 20/PBS, blocked with MicroImmune Blocking Solution (3−4 h at 37 °C in a moist box), aspirated, dried overnight at 37 °C and stored dry at 4 °C in sealed pouches with desiccant. The assay was carried out by simultaneous addition of 25 μl of sera and 75 μl of recombinant anti-RBD neutralising monoclonal antibody (MAB12444; Native Antigen, Oxford, UK) conjugated with HRP, diluted in conjugant buffer (Conjugant Diluent; ClinTech, Guildford, UK) supplemented with 10% FBS and 10% normal human plasma to each well. Plates were incubated for 1 h at 37 °C then washed five times with wash buffer (ClinTech, Guildford, UK). One hundred microlitres of 3,3’,5,5’-Tetramethylbenzidine substrate was then added (ClinTech, Guildford, UK), incubated for 30 min at 37 °C, after which the reaction was stopped and read spectrometrically at 450−630 nm. Results were calculated as a percentage of the average optical density (OD) obtained for three negative controls assayed in each run. Prior to use in this study, the inhibition assay was first validated using the first WHO international reference standard (National Institute for Biological Standards and Control code 20/136) for anti-SARS-COV-2 immunoglobulin. The preparation is used as an internal reference reagent for harmonising such assays^25^. The assigned potency of the standard is 250IU/ampoule, with an arbitrary unitage of 1000 BAU/ml to assist the comparison of assays using anti-RBD IgG^26^. A dilution series was carried out in triplicate and mean values were recorded for percentage inhibition (Supplementary Fig. 3).

### Statistical analysis and reproducibility

Descriptive statistics were used to analyse patient demographics, PCR results and changes in antibody status throughout the study. All statistical tests are two-sided. Paired antibody titres (pre- and post-vaccination) were compared using the Wilcoxon signed-rank test. Association between post-vaccine antibody titre and pre-vaccine seroconversion was assessed with a likelihood ratio test (LRT) on a Tobit censored log-linear regression model for Abbott IgG CMIA antibody titres and with an LRT on a linear regression model for anti-RBD titres. LRTs on multivariable beta regression models were used to assess for an association between the following: RBD antibody-binding inhibition and pre-vaccine seroconversion; RBD antibody-binding inhibition and anti-RBD antibody titre; PsV neutralisation percentage and antibody titres adjusting for pre-vaccine seroconversion. A relationship between post-vaccine inhibition capacity and later infection was assessed using the Wilcoxon rank-sum test. All statistical analyses were carried out using R (Version 4.1.1) with libraries VGAM and betareg. Data utilised to generate results figures are provided (Supplementary Data 1). The source code is available online^27^.

### Ethical approval and consent to participate

Ethical approval of the Sars-Cov-2 antibody response in oLder PEopLe (SCALPEL) study was initially provided by the Chelsea and Westminster NHS Foundation Trust. The proposal underwent review by the Royal Hospital Chelsea Research Oversight Committee. This study was approved by the Health Research Authority and Health and Care Research Wales (IRAS 296291) following a review by the Cambridge Central Research Ethics Committee (Ref.22/EE/0083). All participants provided written informed consent at each sampling interval. PCR testing was carried out as part of routine investigations initiated at the Royal Hospital Chelsea and processing of this patient data has been conducted in line with the Secretary of State’s general notice waiving the requirement of consent for COVID-19 public health, surveillance and research purposes in place at the time of investigation/writing.

### Reporting summary

Further information on research design is available in the Nature Portfolio Reporting Summary linked to this article.

## Results

A total of 280 individuals (median age 83 yrs, IQR 77–89; 95.4% male) participated in the study. Of these, 271/280 participants (96.8%) had received two vaccine doses and 9/280 (3.2%) one vaccine dose by the time of the post-vaccine sero-analysis (Table 1).

**Table 1:** Participant level data for SARS-CoV-2 infection risk and serostatus pre- and post-2 dose Pfizer-BioNTech vaccine regimen.

Characteristic	Overall (*N* = 280)
Age at study start	
Median (Q1, Q3) [min-max]	82 (76, 88) [66-102]
Sex	
Female	13 (4.6%)
Male	267 (95.4%)
Serological evidence of infection before vaccination	
Seronegative (always)	121 (43.2%)
Seropositive (at least once)	153 (54.7%)
[Missing data]	6 (2.1%)
PCR-confirmed infection	
No	181 (64.6%)
Yes	99 (35.4%)
Before June 2020	54
Between June 2020 and 1^st^ vaccine dose	22
Between 1^st^ vaccine dose and April 2021	8
Between April and Oct 2021	15
Reinfection by October 2021	
No	148 (96.7%)
Yes*	5 (3.3%)
Vaccine doses received by April 2021	
One	9 (3.2%)
Two	271 (96.8%)
Evidence of immunosuppression	
Cancer (blood or bone marrow)	6
Splenectomy	1
Receiving methotrexate	1
Receiving apremilast	1
Regular high-dose corticosteroids	1
Severe chronic lung disease	6

*88 patients were unable to be included in the final serology analysis due to death during the study period (25/88), leaving the RHC (7/88), admission to hospital at the time of sampling (6/88), voluntary withdrawal from the study (11/88), becoming an RHC resident prior to the last sample interval only (6/88), pre-vaccine sample spillage (11/88) or insufficient sample volume for all tests (22/88). Participant flow through study available in Supplementary Figure 2.

Column percentages. 88 participants (31.4%) did not have complete antibody test results after vaccination*.Four cases occurred in individuals with prior evidence of seropositivity prior to vaccine and one case with prior seropositivity post single vaccine, none of whom developed severe disease. Four cases developed polymerase chain reaction positive infection at least two weeks post-single vaccine of which all experienced only mild symptoms.

At the time of vaccination, 153 participants had prior evidence of anti-nucleocapsid antibody (anti-NP) and anti-receptor binding domain (RBD) measured by in-house hybrid double antigen-binding assay (DABA) or Abbott SARS-CoV-2 IgG II Quant Assay (Abbott IgG CMIA) seropositivity (baseline proportion = 57.1%). In total, there were 99 polymerase chain reaction (PCR) positive SARS-CoV-2 infections during the study period (Table 1), of which 31 were asymptomatic. Fifteen new cases were seen in the 7 months post-second vaccine. No participants received interleukin-6 inhibitor or combination monoclonal antibody therapy for SARS-CoV-2 infection. While only a small number of participants met the criteria for immunosuppressive conditions, there was no observable difference in antibody titres (Supplementary Fig. 1).

**Fig. 1:**
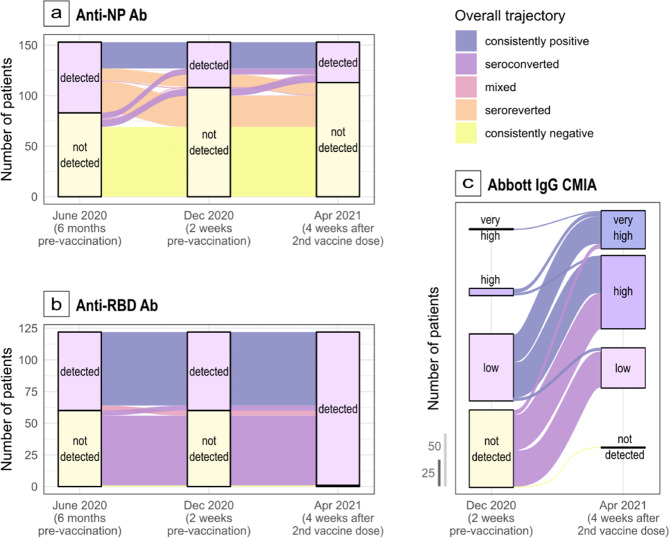
Change in SARS-CoV-2 differential antibody response in an older-adult population across a 10-month period, London, UK, June 2020-April 2021. Vaccination of participants occurred in a two-dose Pfizer-BioNTech vaccine regimen split at 12 weeks and antibodies were then measured at 4 weeks after the second dose. Participants with missing data at any time point were not included in these alluvial plots. **a** *n* = 153 individual participants. Demonstrates change in SARS-CoV-2 anti-NP antibody seropositivity across the cohort measured at three time points between June 2020 and Apr 2021, **b** *n* = 122 individual participants. Demonstrates change in SARS-CoV-2 anti-RBD antibody seropositivity as measured by RBD DABA across the cohort measured at three time points between June 2020 and Apr 2021, **c** *n* = 148 individual participants. Demonstrates change in anti-RBD SARS-CoV-2 IgG antibody seropositivity as quantified by the Abbott SARS-CoV-2 IgG II Quant Assay across the cohort measured at three time points between June 2020 and Apr 2021. Only 54.3% of those initially anti-NP seropositive in June 2020 remained so in December 2020, reducing further to 38.6% by April 2021. In contrast, 93.5% of those seropositive of those anti-RBD seropositive in June remained so 6 months later in December 2020. Post-vaccine (April 2021) anti-RBD seropositivity increased to 98.9%.

Across the study period, anti-NP antibodies appeared to wane at a greater rate than anti-RBD antibodies. Of those individuals with complete data across the whole study, only 38/70 (54.3%) anti-NP antibody seropositive individuals on initial testing remained so at six months compared with 58/62 (93.5%) anti-RBD antibody seropositive individuals (Fig. 1a, b). Post-vaccine anti-NP antibodies continued to wane, with 27/70 (38.6%) remaining seropositive by 10 months (blue flow, Fig. 1a). Post-vaccine 183/185 (98.9%) individuals tested positive for anti-RBD antibodies by RBD DABA and 190/192 (99.0%) tested positive by Abbott IgG CMIA (Fig. 1b, c).

### Pre- and post-vaccine antibody response

Full data were available for primary objective analysis in 192 participants (pre- and post-vaccination). The remaining participants with missing data were unable to be included in the serology analysis due to death during the study period (25/88), leaving the RHC (7/88), admission to hospital at the time of sampling (6/88), voluntary withdrawal from the study (11/88), becoming an RHC resident prior to the last sample interval only (6/88), pre-vaccine sample spillage (11/88) or insufficient sample volume for all tests (22/88) (Supplementary Fig. 2).

**Fig. 2:**
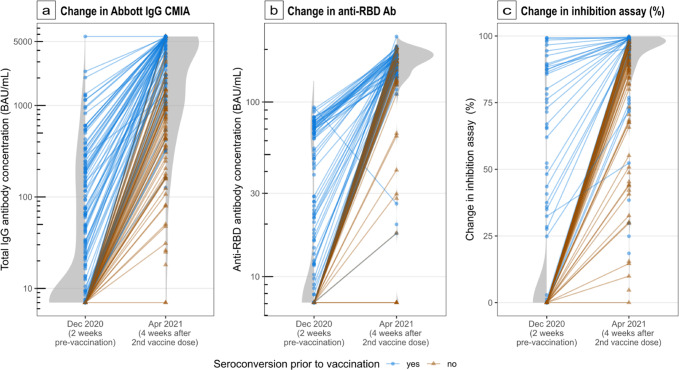
Distributions and change in paired SARS-CoV-2 antibody titres and inhibition capacity pre- and post-vaccination with a two-dose Pfizer-BioNTech BNT162b2 regimen in a population of older adults, London, UK, 2021. The second dose of vaccine was delivered at 12 weeks and antibodies measured 4 weeks later. Results are stratified according to evidence of prior seroconversion (blue) or evidence of seronegative status at baseline (brown). The y-axis (**a**, **b**) follows a logarithmic scale. **a** *n* = 148 individual participants. Quantified antibody titres targeting the S1 subunit of the RBD were calculated using the Architect IgG Quant II CMIA. Data were censored at <7.1 BAU/ml (reported as not detected) and >5680 BAU/ml (above the upper limit of assay detection). **b** *n* = 140 individual participants, anti-RBD antibody titres calculated by in-house hybrid double antigen-binding assay (DABA). **c** *n* = 89 individual participants, change in RBD antibody-binding inhibition for 151/192 patients with sufficient sample for testing.

Post-vaccination, there was a substantial increase in Abbott IgG CMIA titres (increase in median values from 8 to 2150 binding antibody units per ml [BAU/ml]) and RBD DABA antibody (increase in median values from 1 to 177 BAU/ml) concentration (Fig. 2a, b). Using an Abbott titre threshold of >900 BAU/ml as ‘high’, the proportion of participants with ‘high’ Abbott IgG CMIA concentrations increased from 4.9% before vaccination to 72.4% at 4 weeks post-second vaccine (delivered 12 weeks apart).

**Fig. 3:**
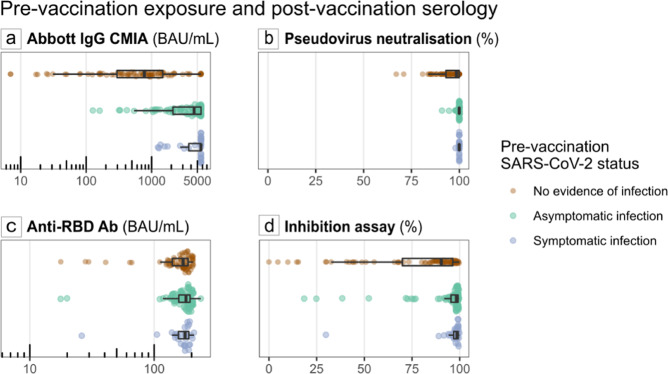
Comparison of antibody titre, pseudovirus neutralisation and inhibition capacity in individuals with and without prior evidence of SARS-CoV-2 infection in an older-adult two-dose vaccinated population. Centre line: median; box limits: first and third quartile; whiskers: 1.5*IQR; points: outliers. Anti-RBD anti-receptor binding domain antibody as measured by double antigen-binding assay. Results sampling post-second dose vaccine stratified by those with no evidence of prior infection (brown), evidence of asymptomatic infection (green) and symptomatic infection (blue). **a** *n* = 192 individual participants. Those with asymptomatic infection saw a 7.5-fold (95% CI 4.6–12.1) increase in Abbott IgG CMIA antibody titres, while those with prior symptomatic infection saw a 20.3-fold (95% CI 9.7–42.5) increase. Data from this study provide strong evidence for these associations in this age group (LRT on Tobit model, *p* < 0.000001). **b** *n* = 184 individual participants. There is very strong evidence for an association between pre-vaccination SARS-CoV-2 exposure and higher neutralisation percentage at time point three (post-second dose vaccine [*p* < 0.0001]), **c** *n* = 182 individual participants. The RBD DABA titre at 4 months post first dose (i.e. 2 weeks after the second dose) is likely to be higher in those who had symptomatic Covid-19 prior to vaccination (95% CI 0.97–1.7; point estimate 1.27) and it is 1.29 times higher in those who only had prior seropositivity (95% CI 1.1–1.6), when compared to those who did not have Covid-19 or seropositivity. Data from this study provide some statistical evidence for these associations (LRT *p* = 0.03). **d** *n* = 183 individual participants. There is very strong evidence for an association between pre-vaccination SARS-CoV-2 exposure and higher RBD antibody-binding inhibition percentage at time point three (*p* < 0.0001).

We found 98/139 (70.5%) participants with high Abbott IgG CMIA antibody concentration after vaccination had prior evidence of seroconversion at the time of vaccination. In contrast, only 7/53 (13.2%) participants with undetectable or low concentrations of Abbott IgG CMIA antibody had evidence of prior seroconversion. Abbott IgG CMIA Abbott IgG CMIA antibody concentration groups did not vary depending on age (ANOVA, *p* = 0.3). The Abbott IgG CMIA antibody titre at 4 weeks after the second vaccine dose (with 12 weeks between the first and second dose) is, on average 9.2-fold higher in people who had previous serological evidence of COVID-19 infection compared to those seronegative throughout (95% CI 5.8–14.5); there is very strong evidence for this effect in this population (likelihood ratio test [LRT] on Tobit model, *p* < 0.00001) (Fig. 2a). Those with asymptomatic infection saw a 7.5-fold (95% CI 4.6–12.1) increase in Abbott IgG CMIA antibody titres while those with prior symptomatic infection saw a 20.3-fold (95% CI 9.7–42.5) increase (Fig. 3a). Data from this study provide strong evidence for these associations in this age group (LRT on Tobit model, *p* < 0.000001).

People with pre-vaccination seropositivity had a higher RBD DABA antibody titre following the second vaccine dose, compared to those without prior seroconversion (+19 BAU/mL; 95% CI: +6 to +32 BAU/mL, linear regression LRT *p* = 0.005) (Fig. 3b). Both RBD DABA and Abbott IgG CMIA titres significantly increased post-administration of two-dose Pfizer-BioNTech vaccine regimens in this population (Wilcoxon signed-rank test, *p* < 0.00001).

### Neutralising activity of post-vaccine antibody response

All participants with RBD DABA antibody/Abbott IgG CMIA seropositive results post-vaccine had evidence of neutralisation activity on initial 1:40 PsV dilution. There was very strong statistical evidence of an association between increasing antibody titres and increasing neutralisation activity percentages (Fig. 3c), even after adjusting for seroconversion prior to vaccination (LRT on a beta model, *p* < 0.00001).

### Anti-RBD competitive ELISA/inhibition assay testing

**Fig. 4:**
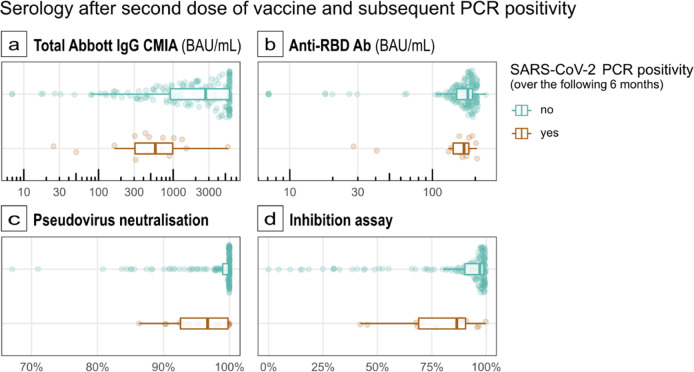
Distribution of anti-RBD antibody titres, neutralisation and inhibition capacity results following a two-dose Pfizer-BioNTech BNT162b2 immunisation compared between those with and without evidence of subsequent infection in an older-adult population. Centre line: median; box limits: first and third quartile; whiskers: 1.5*IQR; points: outliers. SARS-CoV-2 PCR positivity over 6 months post-second dose; no (green), yes (brown) (**a**) *n* = 192 individual participants. Higher IgG titres immediately after vaccination are associated with reduced odds of PCR positivity over the following 6 months. (*p* = 0.002). For each 10-unit increase in Abbott IgG CMIA titre, the odds of PCR positivity decrease by 9% (OR 0.91, 95% CI 0.86–0.97). **b** *n* = 182 individual participants. Data from this study do not provide evidence for a linear association between log anti-RBD titres immediately after vaccination and odds of PCR positivity over the following 6 months (*p* = 0.5). **c** *n* = 184 individual participants Data from this study provide, at best, very weak evidence that a higher pseudovirus neutralisation % immediately after vaccination might be associated with slightly lower odds of PCR positivity over the following 6 months (OR per unit change in neutralisation%: 0.993, 95% CI 0.985 to 1) *p* = 0.07). **d** *n* = 183 individual participants. Data from this study do not provide evidence for a linear association between RBD antibody-binding inhibition % immediately after vaccination and odds of PCR positivity over the following 6 months (*p* = 0.1).

A total of 151/192 participants with paired pre- and post-vaccine samples also underwent testing by anti-RBD antibody competitive enzyme-linked immunosorbent assay (ELISA). A total of 183/192 participants had sufficient samples for post-vaccine testing. Pre-vaccination serological evidence of infection is a strong predictor of higher RBD antibody-binding inhibition after vaccination (beta regression LRT *p* < 0.001 [Fig. 3d]). There is also very strong evidence for an association between increasing anti-RBD concentration and higher RBD antibody-binding inhibition percentage, even after adjusting for pre-vaccination serological evidence of infection (beta regression *p* < 0.001). However, 18 out of 169 (10.7%) participants with a high anti-RBD titre (defined as >100 BAU/mL) have an RBD antibody-binding inhibition of <75%. In our study, post-vaccine median RBD antibody-binding inhibition was 97% among the 168 tested who later did not get infected (IQR: 9%) and 87% in the 15 people who later tested positive (IQR: 22%) (Fig. 4). On univariate analysis (Wilcoxon rank-sum test) there is strong evidence for an association between lower anti-RBD inhibition and subsequent infection (*p* = 0.003).

## Discussion

SARS-CoV-2 continues to represent one of the greatest acute infectious health risks to older people on a global scale. Pre-existing co-morbidities combined with natural immune senescence raise the risk profile of this vulnerable age group and they remain a priority cohort for vaccination^6^. Our findings suggest older adults are able to produce a robust neutralising antibody response following two dose of Pfizer-BioNTech BNT162b2 vaccination among a cohort of individuals intensively iteratively clinically and serologically monitored throughout the pandemic.

Pre-vaccination antibody responses, with a reactive result from at least one assay in individuals with full data available, were detectable across 57.1% (153/274; anti-NP 128 individuals, RBD DABA 128 individuals, Abbott IgG CMIA 105 individuals) of our cohort, despite only 82 positive PCR tests in the same group prior to the first vaccination date. This suggests a potential for considerable asymptomatic spread in this cohort, of which our initial serology data would suggest the majority likely occurred in early March 2020 at the time of the pandemic onset and prior to the wide availability of PCR testing. Hybrid immunity (i.e. those with prior infection) was associated with higher post-vaccine antibody titres, and importantly, this appeared to differ significantly following asymptomatic or symptomatic infection in this group. While all individuals mounting an anti-RBD antibody response here showed some neutralisation activity, our data provides very strong evidence to support booster vaccinations in this cohort with significantly higher post-vaccination anti-RBD antibody titres correlating with increased neutralisation activity. While prior infection alone appears sufficient to induce a detectable antibody response in older adults, this was observed at levels associated with much lower neutralisation activity. Overall infection rates were observed to have considerably reduced after two vaccines. Where breakthrough infections did occur, this was more likely to have occurred in individuals with significantly reduced neutralising activity (Fig. 4). Higher antibody levels alongside less frequent and less severe downstream SARS-CoV-2 infections have also been observed with younger adults^13,28^, and it is encouraging to note similar responses in this vulnerable group especially as recent systematic reviews have commented on inability to reliably control for age^13^. Indeed, Bobrowitz and colleagues comment directly on the lack of data specific to older adults and caution extrapolation of findings in younger adults to guide booster vaccine policy for this group until more data is available^13,14^. How emerging variants of concern, with potential capacity for immune escape, may affect these findings is yet unclear. In the meantime, these findings highlight the vital importance of delivering vaccines to older adults, including those with prior infection, and particularly in communities challenged by limited access.

Recent data has demonstrated that three SARS-CoV-2 spike antigen exposures were required to maximise infection-neutralising capacity, whether by a combination of infection and prime-boost vaccination or by three doses of vaccine in infection naïve individuals^12^. Our data, while conducted in a sub-analysis of our whole cohort, has observed similar findings in an older-adult population, with pseudovirus neutralisation and inhibition measured by competitive ELISA being significantly increased post-two-dose vaccination in those with evidence of prior infection (*p* < 0.001) (Fig. 4). Of note, timing and severity of infection may also play a role in these observations with failure to see any significant boosting of neutralising titres or inhibition when infection and immunisation occurred within a month of each other, while those with COVID-19 pneumonitis appear to mount a higher absolute response (Fig. 3c, d). While we recognise these associations, the small number of cases precludes any further statistical analysis on these particular observations but highlights key areas for investigation going forward. One reason for observing increased inhibition (and reduced incidence of breakthrough infection) in those with three antigen exposures compared to those with only two may be the resultant increase in IgG avidity maturation, long considered necessary to provide protection against reinfection with other viruses^28^. Indeed, recognition of apparent incomplete SARS-CoV-2 antibody avidity maturation in line with waning antibody titres post-infection has previously been raised as concerning potential repeated reinfection^29,30^. In light of these findings, our data suggest that (i) older adults without evidence of natural infection prior to vaccination constitute a particularly important group for third-dose prime-boost vaccination and (ii) require prioritisation of further investigation of the mechanism responsible for observed increased inhibition capacity in those with hybrid infection, which may include the study of antibody avidity maturation kinetics.

Our study is limited by the smaller, predominantly male cohort size when compared to population-based longitudinal studies but is comparatively strengthened by the availability of highly granular data with close follow-up of a relatively isolated community throughout the pandemic. Given that RHC residents are exclusively retired British Army personnel, the majority of participants were male and our data may not be entirely representative of female older adults. The length of our study, and mortality in pre-vaccination SARS-CoV-2 infection, has meant we have been unable to report a full data set for all participants throughout. Where the inhibition assay was developed during the study period, analysis of all samples was limited by consent for further testing, although a suitable proportion of post-vaccine samples were available to allow observations to be made. We recognise the potential limitations in using a PsV, rather than a live virus, assays to assess neutralisation activity, although the comparable safety and versatility of PsV assays make them an attractive alternative to improve access to assessment of neutralising antibody activity^31^. While we have been able to demonstrate the dynamics of antibody response to natural infection and vaccination in this group, we have not conducted assessment of T cell activity which is likely to play a substantial role in the longevity of the immune response. While the vast majority of participants with evidence of natural infection were assessed by inhibition assay no sooner than 6 months after infection, there were a small number of individuals (testing positive between the first and second dose vaccine) that were then assessed at an earlier stage. It is possible that assessment of RBD antibody-binding inhibition for these individuals may have increased given more time and warrants further investigation at a later time point in order to further understand variability in SARS-CoV-2 antibody kinetics. Finally, the effect of three vaccine exposures, as has now routinely been advised in this population, remains unknown. Further investigation will be required to determine if this provides a similar response to three mixed-antigen exposures in older adults.

### Summary

Older adults are capable of mounting a robust antibody response to a two-dose Pfizer-BioNTech BNT162b2 vaccination regimen that was associated with a lower likelihood of post-vaccine infection. Evidence of prior natural infection was associated with increased neutralising activity, increased RBD antibody-binding inhibition and lower incidence of infection, highlighting the importance of considering both antibody quantity and quality when considering correlates of protection. Our findings suggest the potential added benefit of measuring inhibition over antibody titres alone when assessing the protective effect of hybrid immunity in this age group. Our data also provide evidence to support the benefits of an intensified vaccine regimen in this age group. Further evaluation will be required following a third vaccine dose in the context of emerging variants of concern.

## Supplementary information

Supplementary Information

Supplementary Data 1

Description of Additional Supplementary Files

Reporting Summary

## Data Availability

A copy of the source data used in the analysis and development of the figures has been provided with the supplementary data files (Supplementary Data 1). Further information is available from the corresponding author (SJCP; scott.pallett@nhs.net) on reasonable request, as long as this meets local ethical and research governance.

## Code availability

A copy of the custom code (R) is available (https://zenodo.org/record/7843898 (10.5281/zenodo.7843898)^31^. Further information is available via the lead statistician (AM; amazzell@sgul.ac.uk) on reasonable request, as long as this meets local ethical and research governance.
