# Supplementary material for: Hybrid immunity in older adults is associated with reduced SARS-CoV-2 infections following BNT162b2 COVID-19 immunisation
Source: Commun Med (Lond). 2023 Jun 16;3:83. doi: 10.1038/s43856-023-00303-y (PMC10275930; doi:10.1038/s43856-023-00303-y)
Supplement: Supplementary file 1 — Supplementary Information [file 43856_2023_303_MOESM1_ESM.pdf]

Supplementary Information

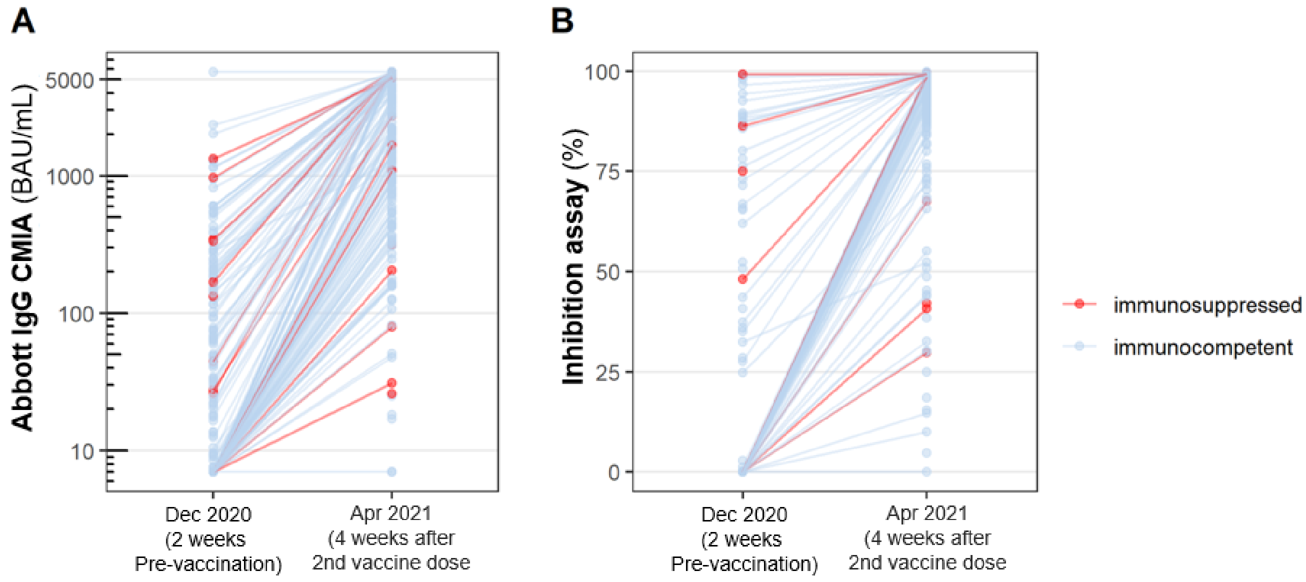

Supplementary Figure 1. Observed change in Abbott SARS-CoV-2 IgG II Quant assay titre and in-house anti-RBD antibody inhibition capacity pre- and post-2-dose prime-boost Pfizer-BioNTech BNT162b2 vaccination, stratified for those with and without documented immunosuppression. *n*=205 individual patients. Those with documented immunosuppression conditions are represented in red and those without in blue.

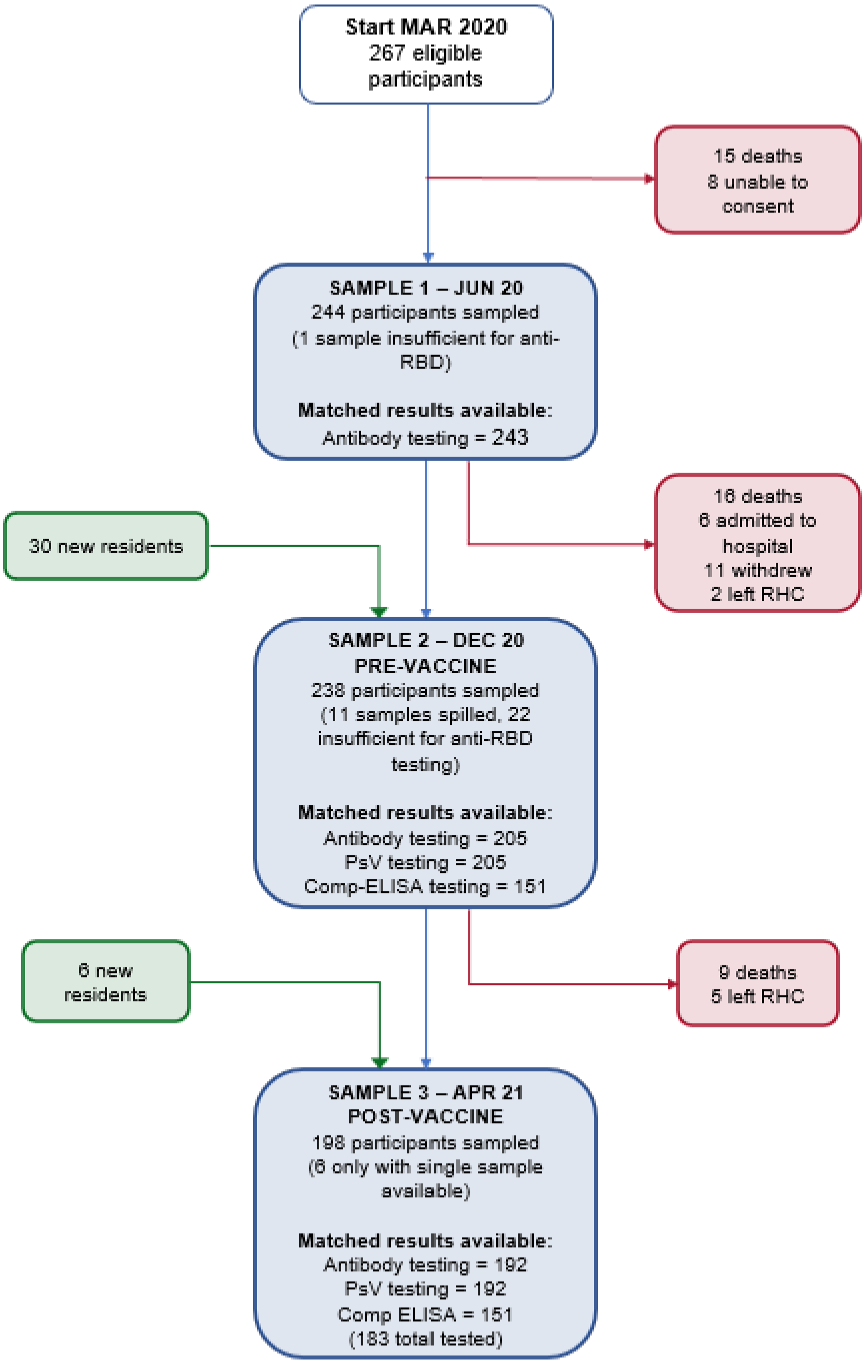

Supplementary Figure 2. Participant flow chart demonstrating sampling pathway and reasons for any loss to follow up. The central blue column shows participant flow through the study for those that were able to undergo sampling at each stage. For post-vaccine matched samples to count towards the overall tally, the participant needed to have also had pre-vaccine sampling. On the right-hand side in red boxes, reasons for participant loss to follow up are presented. On the left-hand side in green boxes, reasons for additional participant recruitment are provided. In total, 280 participants had at least one serology test throughout the study with 192 remaining at study end for overall

analysis. With regards the competitive ELISA/inhibition assay which was developed later in the study, testing was limited to (i) participants that had provided express written consent for any future developed SARS-CoV-2 serology assay and (ii) had sufficient remaining sample. Of the 192 participants at the end of the study, 151 (78.6%) had remaining sufficient sample both pre- and post-vaccine, with a further 32 (total 183, 95.3%) participants having remaining sufficient sample for post-vaccine testing only. The difference (*n* = 9, 4.7%) had not provided consent for this additional test.

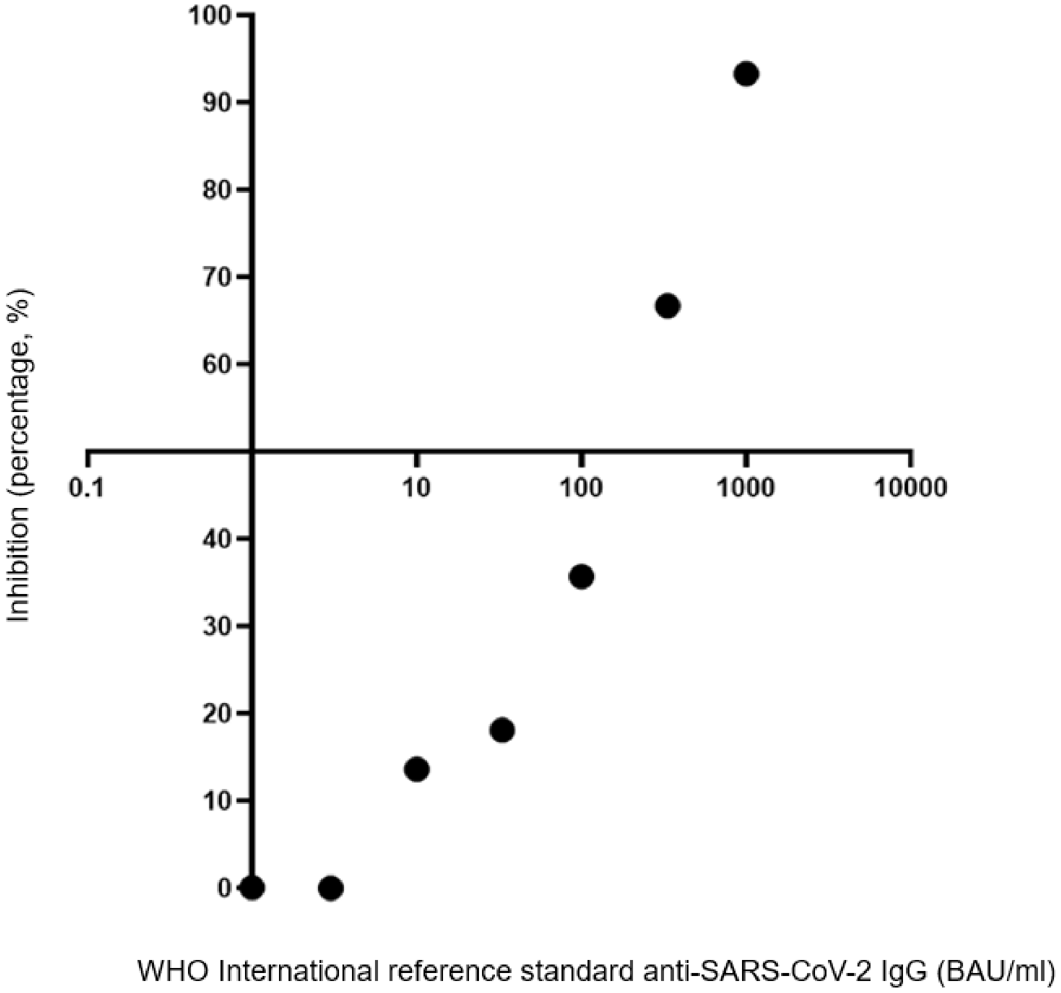

Supplementary Figure 3. Validation data of competitive ELISA showing percentage inhibition against the first World Health Organisation international reference standard for anti-SARS-CoV-2 immunoglobulin. Dilutions were conducted in triplicate starting at 1000BAU/ml (93.33% inhibition), 333 BAU/ml (66.75%), 100 BAU/ml (35.71), 33 BAU/ml (18.14%), 10 BAU/ml (13.62%), 3 BAU/ml (0%), 1 BAU/ml (0.07%).
