# Supplementary material for: Hybrid immunity in older adults is associated with reduced SARS-CoV-2 infections following BNT162b2 COVID-19 immunisation
Source: Commun Med (Lond). 2023 Jun 16;3:83. doi: 10.1038/s43856-023-00303-y (PMC10275930; doi:10.1038/s43856-023-00303-y)
Supplement: Supplementary file 3 — Description of Additional Supplementary Files [file 43856_2023_303_MOESM3_ESM.pdf]

Description of Additional Supplementary File

File Name: Supplementary Data 1

Description: Source data for SCALPEL (Sars-Cov-2 Antibody response in oLder PEopLe) study utilized for analyses and development of figures
