# Supplementary material for: Hybrid immunity in older adults is associated with reduced SARS-CoV-2 infections following BNT162b2 COVID-19 immunisation
Source: Commun Med (Lond). 2023 Jun 16;3:83. doi: 10.1038/s43856-023-00303-y (PMC10275930; doi:10.1038/s43856-023-00303-y)
Supplement: Supplementary file 4 — Reporting Summary [file 43856_2023_303_MOESM4_ESM.pdf]

nature portfolio

Corresponding author(s): Scott Pallett

Last updated by author(s): Apr 18, 2023

Reporting Summary

Nature Portfolio wishes to improve the reproducibility of the work that we publish. This form provides structure for consistency and transparency in reporting. For further information on Nature Portfolio policies, see our Editorial Policies and the Editorial Policy Checklist.

Statistics

For all statistical analyses, confirm that the following items are present in the figure legend, table legend, main text, or Methods section.

n/a	Confirmed	
☐	☒	The exact sample size (*n*) for each experimental group/condition, given as a discrete number and unit of measurement
☒	☐	A statement on whether measurements were taken from distinct samples or whether the same sample was measured repeatedly
☐	☒	The statistical test(s) used AND whether they are one- or two-sided<br>*Only common tests should be described solely by name; describe more complex techniques in the Methods section.*
☐	☒	A description of all covariates tested
☒	☐	A description of any assumptions or corrections, such as tests of normality and adjustment for multiple comparisons
☐	☒	A full description of the statistical parameters including central tendency (e.g. means) or other basic estimates (e.g. regression coefficient) AND variation (e.g. standard deviation) or associated estimates of uncertainty (e.g. confidence intervals)
☐	☒	For null hypothesis testing, the test statistic (e.g. *F*, *t*, *r*) with confidence intervals, effect sizes, degrees of freedom and *P* value noted<br>*Give P values as exact values whenever suitable.*
☒	☐	For Bayesian analysis, information on the choice of priors and Markov chain Monte Carlo settings
☒	☐	For hierarchical and complex designs, identification of the appropriate level for tests and full reporting of outcomes
☐	☒	Estimates of effect sizes (e.g. Cohen's *d*, Pearson's *r*), indicating how they were calculated

Our web collection on statistics for biologists contains articles on many of the points above.

Software and code

Policy information about availability of computer code

Data collection	Microsoft Excel.
Data analysis	R (Version 4.1.1) with libraries VGAM and betareg.

For manuscripts utilizing custom algorithms or software that are central to the research but not yet described in published literature, software must be made available to editors and reviewers. We strongly encourage code deposition in a community repository (e.g. GitHub). See the Nature Portfolio guidelines for submitting code & software for further information.

Data

Policy information about availability of data

All manuscripts must include a data availability statement. This statement should provide the following information, where applicable:

- Accession codes, unique identifiers, or web links for publicly available datasets
- A description of any restrictions on data availability
- For clinical datasets or third party data, please ensure that the statement adheres to our policy

A copy of source data used in the analysis and development of the figures has been provided with the supplementary data files. Further information is available from the corresponding author (SJCP; scott.pallett@nhs.net) on reasonable request, as long as this meets local ethical and research governance.

Human research participants

Policy information about studies involving human research participants and Sex and Gender in Research.

Reporting on sex and gender	Of the 280 participants in the study, 95.4% were male (both assigned and self-reported). The entire population of the study were made up of veteran Chelsea Pensioners and were therefore skewed towards male sex based on historical demographic make up of the British Army. This is commented on in the manuscript and raised as a potential limitation.
Population characteristics	Of the 280 participants in the study the median age was 83 years, IQR 77-89, reflecting the target high-risk for severe SARS-CoV-2 infection study population of older adults residing in long-term care facilities. Relevant chronic disease and immune suppressive conditions, as defined by the UK Health Security Agency advice on high-risk conditions for SARS-CoV-2, have been taken into account in the analysis which is presented in the manuscript.
Recruitment	All Royal Hospital Chelsea residents throughout the duration of the reporting period, and were able to provide written informed consent, were eligible for inclusion in the study. Participants were excluded from the study if they were unable to provide written informed consent, prior recruitment to the UK Health Security Agency supported Sarscov2 Immunity& REinfection EvalutioN (SIREN) study or receipt of a vaccine other than the Pfizer BioNTech BNT162b2.
Ethics oversight	Ethical approval of the Sars-Cov-2 Antibody response in oLder PEopLe (SCALPEL) study was initially provided by the Chelsea and Westminster NHS Foundation Trust. The proposal underwent review by the Royal Hospital Chelsea Research Oversight Committee. This study was approved by the Health Research Authority and Health and Care Research Wales (IRAS 296291) following a review by the Cambridge Central Research Ethics Committee (Ref.22/EE/0083). All participants provided written informed consent at each sampling interval. PCR testing was carried out as part of routine investigations initiated at the Royal Hospital Chelsea and processing of this patient data has been conducted in line with Secretary of State general notice waiving requirement of consent for COVID-19 public health, surveillance and research purposes.

Note that full information on the approval of the study protocol must also be provided in the manuscript.

Field-specific reporting

Please select the one below that is the best fit for your research. If you are not sure, read the appropriate sections before making your selection.

☒ Life sciences ☐ Behavioural & social sciences ☐ Ecological, evolutionary & environmental sciences

For a reference copy of the document with all sections, see nature.com/documents/nr-reporting-summary-flat.pdf

Life sciences study design

All studies must disclose on these points even when the disclosure is negative.

Sample size	It was possible to invite the entire target population (Royal Hospital Chelsea residents) to participate in the study and the overwhelming majority (>95%) agreed; therefore, sampling was not required. The main study endpoint use quantitative outcomes, for which data from 280 participants are sufficient.
Data exclusions	No data were excluded from the analyses.
Replication	All assays used in the study underwent extensive verification, including sample result reproducibility, with references provided for prior verification studies within the manuscript. Further replication of sample testing within the study was not undertaken due to small volume single time point samples taken in line with the ethics approval.
Randomization	Randomisation was not appropriate for this study, with all individuals receiving at least one dose of SARS-CoV-2 Pfizer BionTech BNT162b2 vaccine as a prerequisite of the study.
Blinding	All investigators were blinded to participant (i) prior infection status (ii) prior serological status (iii) demographic data and (iv) history of immune suppressive conditions at all stages of the study.

Reporting for specific materials, systems and methods

We require information from authors about some types of materials, experimental systems and methods used in many studies. Here, indicate whether each material, system or method listed is relevant to your study. If you are not sure if a list item applies to your research, read the appropriate section before selecting a response.

Materials & experimental systems

n/a	Involved in the study
☒	☐ Antibodies
☒	☐ Eukaryotic cell lines
☒	☐ Palaeontology and archaeology
☒	☐ Animals and other organisms
☐	☒ Clinical data
☒	☐ Dual use research of concern

Methods

n/a	Involved in the study
☒	☐ ChIP-seq
☒	☐ Flow cytometry
☒	☐ MRI-based neuroimaging

Clinical data

Policy information about clinical studies

All manuscripts should comply with the ICMJE guidelines for publication of clinical research and a completed CONSORT checklist must be included with all submissions.

Clinical trial registration: Registered on NHS Health Research Authority. Integrated Research Application System (IRAS) ID: 296291.

Study protocol: Research square

Data collection: All sample and data collection were conducted at the Royal Hospital Chelsea. Informed written consent was provided prior to any individual samples being taken at each round. Participants entered the study Dec 2020, with sampling prior to the first vaccine dose and continued, unless opting to withdraw, until a second sample was taken 4 weeks after the second vaccine dose following repeat written informed consent.

Outcomes: Pre-defined primary outcomes was the ability of older adults to mount a robust humoral immune response, as defined by detection of antibody titre and neutralisation activity in the context of immunisation with or without prior infection. Secondary outcomes looked to establish potential correlates of protection through measurement of antibody inhibition capacity and correlation with subsequent infection as established by weekly SARS-CoV-2 PCR testing in a closed environment supported by SARS-CoV-2 anti-nucleocapsid antibody testing. Where available commercial assays were utilised or inhouse assays developed.
